# The swedish paediatric JIA-registry

**DOI:** 10.1186/1546-0096-12-S1-P5

**Published:** 2014-09-17

**Authors:** Bo Magnusson

**Affiliations:** 1 Paediatric Rheumatology Unit, Astrid Lindgren Children´s hospital, Karolinska University Hospital, Stockholm, Sweden

## Introduction

The Swedish JIA-registry started in 2009 with the primary goal to follow all children on biologics, cytokine modulators, but later expanded to all patients with or without antirheumatic drugs. It offers care givers a tool for overview on patient level in clinical settings and involves patients and parents as partners in the process. Patient reports are given through e-tablets and summaries will be provided as feed backs. Diagrams on treatment between regions can be followed on line and data can be extracted to promote quality work in the local interprofessional team. National data can be analyzed together with official registries with the aim to follow future morbidity and process of care given to patients with the aim for equal care for all patients. Eye examinations and uveitis is integrated in the registry.

## Objectives

1. Description of the registry

2. Registration rates and coverage

3. Patterns of medical treatmen

## Methods

Use of web-based national registry with reports from care givers, patients and medical records. JADAS, CHAQ, Disabkids including the arthritis specific questions are followed together with growth.

## Results

After 5 years there are 1700 patients included and data enough to make analyzes possible. Coverage is almost complete but registration rates differ between regions. The total registration rate is above 60 % for all JIA and above 90 % for patients on cytokine modulators. The figures are calculated from prevalence date in cohort studies done together with data from the official patient registry of care given in Sweden for JIA and data from register for over the counter sell of cytokine modulators. The use of cytokine modulators differs from 15 up to 35 patients / 100 000 between different care givers. Some of the drugs are not approved for use in children and are given in schedules not recommended. Guidelines for treatment are not fully followed. Methotrexate as single treatment is used as often as cytokine modulators. Up to 32 % of the patients are on no antirheumatic or continuous NSAID treatment. Mean JADAS-27 for all JIA is 15 at onset going down to 5 after 2 years and staying at that level. Most of JADAS is explained from patient report of health and not from active disease. Most patients have no arthritis at latest registration. It is a challenge to improve health and not just freedom of disease activity.

## Conclusion

Registration rates and number of included patients are high enough to make it possible to start analyzing data from the registry. There are differences between regions in all aspects of treatment but so far no data showing differences of outcome or health which cannot be explained by differences in reporting data or differences in registration rates. The registry is a tool for care givers in partnership with patients and gives data for local quality work. There is a need for treatment goals, key ratios and treat to target definitions in different subgroups of JIA.

## Disclosure of interest

None declared.

